# Chain management reduces inflammation (IL-1b, IL-6, IL-8, TNF-a, PCT) and improves vascular (NO, DD, VWF, and ET-1)/immune function in acute respiratory failure: A retrospective cohort study

**DOI:** 10.5937/jomb0-58099

**Published:** 2025-10-28

**Authors:** Yunxia Chen, Xinyu Yuan, Sisi Sun, Wenjuan Ding, Ningning Dai, Jie Wang

**Affiliations:** 1 The Fourth Affiliated Hospital of Soochow University, Department of Respiratory and Critical Care Medicine, Suzhou Dushu Lake Hospital, Suzhou, Jiangsu, 215125, China; 2 The Fourth Affiliated Hospital of Soochow University, Department of Emergency Medicine, Suzhou, Jiangsu, 215125, China; 3 The Fourth Affiliated Hospital of Soochow University, Department of Emergency Medicine, Suzhou Dushu Lake Hospital, Suzhou, Jiangsu, 215125, China; 4 The Fourth Affiliated Hospital of Soochow University, Department of Nursing, Suzhou Dushu Lake Hospital, Suzhou, Jiangsu, 215125, China

**Keywords:** acute respiratory failure, inflammatory factors, vascular endothelial function, T lymphocyte subsets, akutna respiratorna insuficijencija, inflamatorni faktori, funkcija vaskularnog endotela, T-limfocitne podgrupe

## Abstract

**Background:**

Given the critical importance of inflammation, immune and endothelial function in acute respiratory failure (ARF), it is essential to evaluate therapeutic strategies that target these pathways to confirm their application value. This study aimed to investigate the impact of chain management (defined as a systematic protocol integrating dynamic risk assessment, standardised nursing pathways, multidisciplinary coordination, and real-time biomarker monitoring to optimise clinical decision-making) on inflammatory markers (interleukin [IL]-1b, IL-6, IL-8, tumour necrosis factor[TNF]-a, and procalcitonin[PCT]), vascular endothelial function, blood gas parameters, and T lymphocyte subsets in patients with ARF.

**Methods:**

A retrospective analysis was conducted on 101 ARF patients admitted between October 2023 and December 2024. The patients were categorised into two groups: a conventional group (55 cases, receiving standard risk warning management) and a chain group (46 cases, undergoing chain management). Levels of inflammatory factors and vascular endothelial markers (nitric oxide[NO], endothelin-1 [ET-1], etc.) were measured using enzyme-linked immunosorbent assay (ELISA), blood gas function was evaluated with a blood gas analyser, and T lymphocyte subsets (CD3+, CD4+, and CD8+) were analysed via flow cytometry.

**Results:**

Compared to the conventional group, the chain group demonstrated significantly shorter durations of mechanical ventilation and ICU stays (P&lt;0.05). Moreover, the chain group exhibited more pronounced reductions in inflammatory factors, including IL-1b, TNF-a, and PCT (P&lt;0.05). Improvements in vascular endothelial function were also more evident in the chain group, with higher NO levels and lower ET-1 levels (P&lt;0.05). Additionally, the chain group achieved better blood gas outcomes, characterised by higher PaO2 and lower PaCO2 levels (P&lt;0.05), as well as greater increases in CD3+ and CD4+ cell counts (P&lt;0.05).

**Conclusions:**

Chain management effectively mitigates inflammatory responses and enhances vascular immune function, endothelial function in ARF patients through multi-targeted interventions.

## Abstract (sr)

**Metode:**

Retrospektivna analiza je sprovedena na grupi od 101 pacijenta sa ARF-om, primljenih između oktobra i decembra 2024. Pacijenti su podeljeni u dve grupe: konvencionalnu grupu (55 slučajeva, sa standardnim upravljanjem rizikom) i lančanu grupu (46 slučajeva, sa primenom upravljanja lancem). Nivoi inflamatornih faktora i markera vaskularnog endotela (azot-oksid [NO], endotelin-1[ET-1] i dr.) su mereni pomoću ELISA metode; funkcija gasne razmene u krvi procenjena je analizatorom gasova, a T-limfocitne podgrupe (CD3+, CD4+ i CD8+) analizirane su protočnom citometrijom.

**Rezultati:**

U poređenju sa konvencionalnom grupom, lančana grupa je pokazala značajno kraće trajanje mehaničke ventilacije i boravka u jedinici intenzivne nege (P&lt;0,05). Takođe, zabeleženo je izraženije smanjenje inflamatornih faktora kao što su IL-1b, TNF-a i PCT (P&lt; 0,05). Poboljšanja u funkciji vaskularnog endotela bila su izraženija u lančanoj grupi, sa višim nivoima NO i nižim nivoima ET-1 (P &lt; 0,05). Pored toga, lančana grupa je imala bolje rezultate gasne razmene, karakterisane višim nivoima PaO2 i nižim nivoima PaCO2 (P &lt; 0,05), kao i veće poraste broja CD3+ i CD4+ ćelija (P &lt; 0,05).

**Zaključak:**

Upravljanje lancem efikasno ublažava inflamatorni odgovor i poboljšava vaskularnu i imunološku funkciju, kao i endotelnu funkciju kod pacijenata sa ARF putem višeciljanih intervencija.

## Introduction

Acute respiratory failure (ARF), characterised by inadequate oxygenation (hypoxemia) or insufficient ventilation (hypocapnia), represents a prevalent clinical condition with significant morbidity and mortality [1]. Commonly precipitated by conditions such as pneumonia and chronic obstructive pulmonary disease (COPD), ARF poses a substantial burden on healthcare systems [2]. The COVID-19 pandemic has further exacerbated this issue, as viral pneumonia has led to a marked increase in ARF cases. Data from 2020 indicate that 5%–10% of COVID-19 patients progress to severe ARF, highlighting the urgency of effective management strategies [3]. Although this study did not include COVID-19 patients, the inflammatory mechanisms explored may be relevant to viral-induced ARF.

The pathophysiology of ARF is known to be closely linked to acute pulmonary inflammation and vascular endothelial injury [4]. For example, Renieris et al. highlighted the role of interleukin (IL)-1 in driving tissue-specific inflammation and severe respiratory failure in COVID-19 patients [5]. Similarly, Akgün Ünlü et al. [6] established a correlation between elevated IL-8 levels and increased mortality in pediatric ARF cases through experiments. Furthermore, Herrera et al. [7] identified the endothelin-1 (ET-1)/vascular endothelial growth factor (VEGF) signalpeptide receptor as a promising therapeutic target for acute respiratory distress syndrome. These studies collectively emphasise the central role of inflammatory responses and vascular endothelial dysfunction in the progression of ARF.

Given the critical importance of inflammation and endothelial function in ARF, it is essential to evaluate therapeutic strategies that target these pathways to confirm their application value. This study assesses the impact of chain management on ARF, with a particular focus on key biomarkers such as IL-1β, IL-8, and ET-1. It aims to provide valuable insights and evidence-based recommendations for optimising ARF treatment in the future.

## Materials and methods

### Study subjects

This retrospective analysis included patients diagnosed with ARF who were admitted between October 2023 and December 2024. The required sample size was estimated using PASS software (α = 0.05, effect size = 0.3, test efficacy = 0.8), with a target of 93 cases. From an initial pool of 184 potential participants, 137 patients met the inclusion criteria, which comprised confirmed ARF, eligibility for mechanical ventilation, ICU admission, and availability of complete clinical data. After applying exclusion criteria, such as the presence of malignancies, hematologic disorders, speech, cognitive or hearing impairments, psychiatric conditions, immune dysfunction, or impaired consciousness, the final study cohort consisted of 101 patients. Patients were retrospectively categorised into groups based on adherence to the risk-warning protocol during their hospitalisation. These patients were divided into two groups: 55 patients who received conventional risk warning management (conventional group) and 46 patients who underwent chain management (chain group). The final cohort included 101 patients (55 vs. 46) due to incomplete clinical records in 36 excluded cases, predominantly from the conventional group.

### Ethical statement

The study protocol was approved by our institution’s ethics committee and conducted in accordance with the principles outlined in the Declaration of Helsinki. It was a retrospective analysis, and all data were anonymised. The ethics committee deemed the data exempt from informed consent.

### Methods

Conventional group: (1) A dedicated risk warning management team was established to address ARF-related risks. (2) To maintain airway hydration, a humidifier was utilised, and patients were encouraged to increase fluid intake to promote sputum expulsion. Back percussion was performed every two hours to assist patients in effective coughing and sputum clearance. Once the patient’s condition stabilised, appropriate respiratory training was arranged to strengthen their respiratory function.

Chain group: (1) Chain management was integrated with the risk warning system. An ARF clinical nursing intervention table was developed, outlining daily care protocols such as ventilator management, routine sputum clearance, condition monitoring, and adverse event management. Nursing staff adhered strictly to the table to ensure consistent and standardised care delivery. (2) During ventilator-assisted treatment, the position of the ventilator was adjusted according to each patient’s specific needs. Sterile water was added to the humidification chamber to ensure oral moisture and hygiene. Additionally, gastrointestinal decompression was employed to relieve abdominal distension and high-risk infection cases were treated with combined nebulisation therapy to combat infections. (3) Patients were regularly repositioned and received back percussion to facilitate sputum clearance. Techniques for effective coughing were taught to encourage independent sputum expulsion. For patients struggling with sputum clearance, mucolytic nebulisation or negative pressure suction was utilised to maintain airway patency and reduce the risk of respiratory infections. (4) Following the use of ventilators, nebulisers, or sputum suction devices, disposable equipment was disposed of as medical waste. Reusable equipment was thoroughly disinfected, labelled with patient identifiers, and stored separately to minimise the risk of cross-contamination. (5) Patients were advised to follow a highprotein, easily digestible diet consisting of liquid or semi-liquid foods. Increased fluid intake was recommended to help thin sputum and improve clearance. Patients received enteral nutritional support with daily supplementation of omega-3 fatty acids (2 g/d) and immune enhancers. (6) The psychological state of patients was closely monitored. Those displaying significant negative emotions were offered counseling, while individuals at high risk for psychological distress received immediate interventions to address their mental health needs.

Nursing adherence to the chain management protocol was audited weekly, with >90% compliance recorded. Both groups received low tidal volume ventilation (6–8 mL/kg predicted body weight), and PEEP was titrated to maintain PaO_2_/FiO_2_>200 mmHg, with no significant inter-group differences in these parameters.

### Data collection

Baseline data, including age, gender, and disease duration, were systematically recorded.

### Sample collection and testing

Arterial and venous blood samples were collected from patients upon admission and on the first day after ICU discharge. Inflammatory markers, including IL-1β, IL-6, IL-8, tumour necrosis factor (TNF)-α, and procalcitonin (PCT), as well as vascular endothelial function indicators such as nitric oxide (NO), ΔD, von Willebrand factor (vWF), and ET-1, were quantified using ELISA. Blood gas parameters, including the potential of hydrogen (pH), partial pressure of arterial oxygen (PaO_2_), partial pressure of arterial carbon dioxide (PaCO_2_), and arterial oxygen saturation (SaO_2_), were measured using a blood gas analyser. Additionally, T lymphocyte subsets (CD3^+^, CD4^+^, and CD8^+^) were analysed using flow cytometry. ELISA kits (R&D Systems, USA), blood gas analyser (Radiometer ABL90 FLEX, Denmark), and flow cytometer (BD FACSCanto II, USA).

### Statistical analysis

All statistical analyses were performed using SPSS 26.0 software. Data normality was tested using Shapiro-Wilk tests before applying parametric analyses. Continuous variables were expressed as mean ± standard deviation (x̄±s) and compared using independent t-tests. Categorical variables were presented as frequencies and percentages, with comparisons made using chi-square tests. A P-value of less than 0.05 was considered statistically significant.

## Results

### Comparison of baseline characteristics

**Figure 1:**
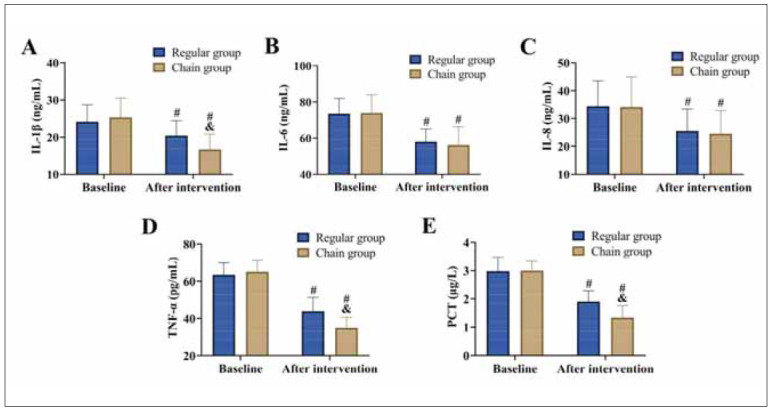
Comparison of inflammatory responses<br>(A) IL-1β, (B) IL-6, (C) IL-8, (D) TNF-α, and (E) PCT. vs. baseline ^#^P<0.05, vs. regular group ^&^P<0.05.

A comparison of baseline characteristics between the two groups revealed no statistically significant differences (*P*>0.05). Furthermore, all standardised mean differences (SMD) were below 0.2, indicating that the groups were well-balanced and comparable. Table 1 Figure 1

**Table 1:** Comparison of baseline characteristics.

Groups		Regular group	Chain group	χ^2^ (t)	P	SMD
Sex	Male	35	27	0.258	0.612	0.134
	Female	20	19			
Age		69.29±3.22	69.65±4.00	0.503	0.616	0.114
Duration of disease (years)		8.51±2.64	7.78±2.08	1.516	0.133	0.184
BMI (kg/m^2^)		25.25±2.55	25.50±2.69	0.479	0.633	0.134
Degree of<br>seriousness	Mild	29	22	1.189	0.552	0.084
	Moderate	25	24			
	Severe	1	0			
Primary disease	Pneumonia	16	16	0.55	0.908	0.116
	Bronchial asthma	25	19			
	COPD	10	7			
	Other	4	4			

### Comparison of inflammatory responses

At baseline, there were no significant differences in inflammatory markers between the two groups (*P*>0.05). Following the intervention, both groups exhibited a marked reduction in levels of IL-1β, IL-6, IL-8, TNF-α, and PCT (P<0.05). Importantly, the chain group achieved significantly lower levels of IL-1β, TNF-α, and PCT compared to the conventional group (*P*<0.05).

### Comparison of vascular endothelial function

**Figure 2:**
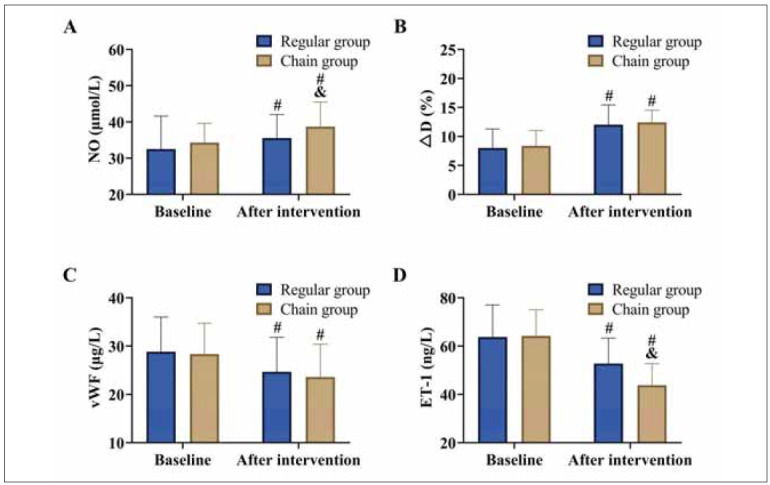
Comparison of vascular endothelial function.<br>(A) NO, (B) ΔD, (C) vWF, and (D) ET-1. vs. baseline ^#^P<0.05, vs. regular group ^&^P<0.05.

**Figure 3:**
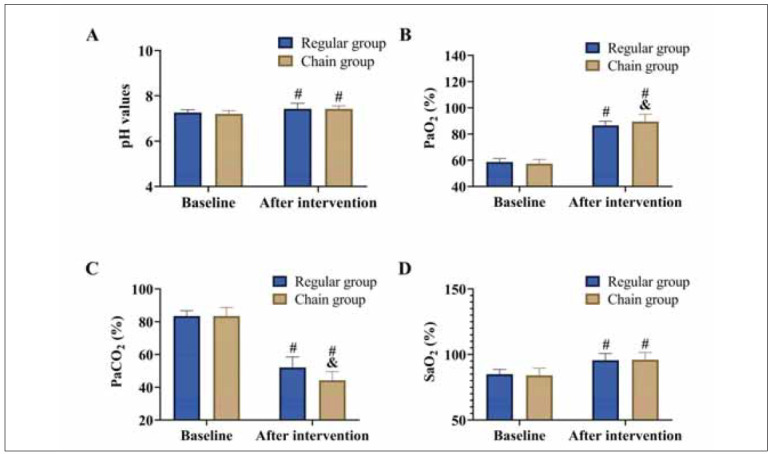
Comparison of blood gas parameters.<br>(A) pH, (B) PaO_2_, (C) PaCO_2_, and SaO_2_. vs. baseline ^#^P<0.05, vs. regular group ^&^P<0.05.

**Figure 4:**
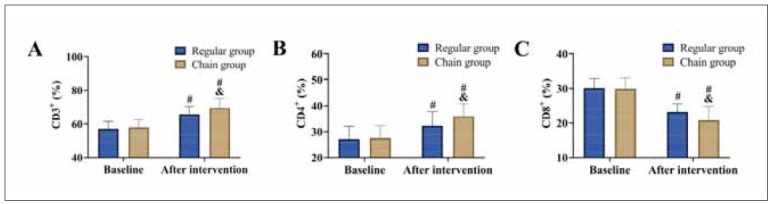
Comparison of T lymphocyte subsets<br>(A) CD3^+^, (B) CD4^+^, and (C) CD8^+^. vs. baseline ^#^P<0.05, vs. regular group ^&^P<0.05.

Baseline measurements of NO, ΔD, vWF, and ET-1 showed no significant differences between the groups (*P*>0.05). Post-intervention, both groups experienced an increase in NO and ΔD levels, alongside a decrease in vWF and ET-1 levels (*P<*0.05). Notably, the chain group outperformed the conventional group, with significantly higher NO levels and lower ET-1 levels compared to the conventional group (*P*<0.05). However, no significant inter-group differences were observed in ΔD and vWF levels (*P*>0.05). Figure 2 Figure 3 Figure 4

### Comparison of blood gas parameters

After the intervention, improvements in blood gas parameters were observed in both groups, with increases in pH, PaO_2_, and SaO_2_ levels, as well as a reduction in PaCO_2_ levels (*P*<0.05). When comparing the two groups, no significant differences were found in pH and SaO_2_ levels (*P*>0.05). However, the chain group achieved higher PaO_2_ levels and lower PaCO_2_ levels compared to the conventional group (*P*<0.05).

### Comparison of T lymphocyte subsets

Finally, the analysis of T lymphocyte subsets revealed that both groups experienced an increase in CD3^+^ and CD4^+^ levels and a decrease in CD8^+^ levels post-intervention (*P*<0.05). Compared to the conventional group, the chain group showed significantly higher CD3^+^ and CD4^+^ levels (P<0.05), while CD8^+^ levels did not differ significantly between the groups (*P*>0.05).

## Discussion

In this study, we observed that chain management played a pivotal role in alleviating inflammatory responses and improving vascular endothelial function in ARF patients. These findings offer new perspectives and potential directions for optimising ARF treatment in the future.

As previously discussed, ARF patients frequently exhibit systemic hyperactivation of inflammatory responses, where an imbalance between pro-inflammatory factors (e.g., IL-6, TNF-α) and anti-inflammatory mediators can precipitate multi-organ dysfunction [8] [9] [10]. In this study, the chain group showed more pronounced improvements in inflammatory responses compared to the conventional group. Several underlying mechanisms may explain these improvements: (1) Dynamic monitoring of vital signs and biomarkers allowed for the timely initiation of anti-infective therapies and immune modulation, which may involve modulation of NF-kB signalling pathways and reducing the release of pro-inflammatory cytokines. (2) The implementation of low tidal volume and limited plateau pressure in mechanical ventilation strategies minimised alveolar overdistension and biotrauma, thereby reducing the release of IL-1β and IL-8 by alveolar macrophages. (3) Rapid microbiological testing and targeted anti-infective treatments accelerated pathogen clearance, preventing the amplification of inflammatory cascades.

Vascular endothelial injury plays a central role in the microcirculatory dysfunction and coagulationabnormalities observed in ARF patients. We hypothesise that chain management enhances endothelial function through real-time adjustments of FiO and PEEP to maintain PaO /FiO >200 mmHg, thereby mitigating hypoxia-induced mitochondrial damage and oxidative stress in endothelial cells. This hypothesis is supported by the observation that the chain group achieved significantly higher PaO and lower PaCO levels post-intervention compared to the conventional group. Furthermore, the early administration of low molecular weight heparin for thrombosis prevention, combined with hemodynamic monitoring, improved the expression of endothelial anticoagulant proteins, significantly reducing ET-1 release [11]. It is also essential to consider the intricate interplay between inflammation and endothelial regulation. It is well-documented that inhibiting inflammatory factors from disrupting endothelial tight junction proteins can decrease vascular permeability and alleviate pulmonary oedema [12]. In this study, the observed reduction in inflammatory responses in the chain group may have indirectly contributed to the greater improvement in vascular endothelial function.

Finally, the analysis of T lymphocyte subsets revealed that the chain group had significantly higherlevels of CD3^+^ and CD4^+^ cells following the intervention. This observed improvement may be linked to several factors, including the regulation of stress hormones, effective infection control, and the implementation of immunonutrition strategies. By optimising analgesia and sedation protocols, chain management helped mitigate excessive cortisol secretion, thereby reversing the Th1/Th2 cell shift toward Th1 dominance and promoting the proliferation of CD4^+^ cells [13] [14]. Furthermore, the early administration of enteral nutrition, such as -3 fatty acids, combined with immune enhancers, enhanced lymphocyte metabolic activity, resulting in elevated CD3^+^ and CD4^+^ cell counts [15] [16].

Despite these promising findings, this study is not without limitations, many of which stem from its retrospective design. The study population was primarily composed of ICU patients, which may restrict the generalizability of the results to other settings or less severe cases. Future research is needed to evaluate the effectiveness of chain management in nonmechanically ventilated patients or those with milder forms of ARF. Additionally, the study did not include long-term follow-up data on critical outcomes such as lung function, quality of life, or readmission rates, warranting subsequent investigations. Another limitation lies in the lack of standardised clinical guidelines for chain management, which may have led to variability in implementation and potentially impacted the comparability of results. Finally, the small sample size of this study (n=101) may limit statistical validity, and future expansion of the sample size is needed to validate the findings.

## Conclusion

Chain management holds significant promise for improving outcomes in ARF patients by targeting early inflammatory cascades, optimising oxygenation and microcirculation, and addressing immune dysregulation. However, its broader application faces several challenges, including the need for deeper mechanistic insights, the heterogeneity of patient populations, and the dependency on resource availability.

## Dodatak

### Availability of data and materials

The data used to support the findings of this study are available from the corresponding author upon request.

### Funding

No funds, grants, or other support were received.

### Acknowledgements

Not applicable.

### Conflict of interest statement

All the authors declare that they have no conflict of interest in this work.
